# Novel variation associated with species range expansion

**DOI:** 10.1186/1471-2148-10-382

**Published:** 2010-12-09

**Authors:** James Buckley, Jon R Bridle, Andrew Pomiankowski

**Affiliations:** 1 University of Bristol, School of Biological Sciences, Woodland Road, Bristol, BS8 1UG, UK; 2 The Galton Laboratory, Department of Genetics, Evolution and Environment, University College London, 4 Stephenson Way, London NW1 2HE, UK; 3 CoMPLEX, University College London, Gower Street, London WC1E 6BT, UK

## Abstract

When species shift their ranges to track climate change, they are almost certain to experience novel environments to which they are poorly adapted. Otaki and co-workers document an explosion of wing pattern variation accompanying range expansion in the pale grass blue butterfly. This pattern can be replicated in the laboratory using artificial selection on cold shocked pupae, at temperature extremes typical of recently colonized environments. We discuss how this phenotypic plasticity may be associated with successful colonization and how significant local adaptation is likely to re-establish developmental control. Integrating knowledge of trait plasticity into current genetic models of adaptation is central to our understanding of when and where a colonising population will be able to persist and adapt in novel surroundings.

## Commentary

Species ranges are never fixed, but remain in continual flux in response to demographic, genetic, ecological and environmental variation. Colonization occurs at the range margin when populations spill over into new sites, typically followed by population extinction as environmental and other forces prevent persistence in these new habitats. As the climate has warmed, this turn-over has resulted in expansions that appear more permanent. Consistent northward range shifts have been documented for several vertebrate and invertebrate species in the northern hemisphere, but particularly Lepidoptera, whose historical distributions are usually well-known [1,2]. But what do colonizers look like in these new populations - are they just a sample of the main population or do they differ phenotypically or genotypically? How do these new populations adapt to the novel environmental conditions they encounter and is change likely to persist over longer time scales?

Joji Otaki and co-workers [1] have charted recent range expansion in the beautifully named pale grass blue butterfly (*Zizeeria maha*). From 1990-2000, this species progressively marched more than 100 km up the west coast of Japan, towards the top of Honshu island - known in precise detail thanks to assiduous collecting of amateur Japanese lepidopterists. Similar northward movements are known in several other Japanese butterflies [1]. Temperature records suggest increasing summer temperatures facilitated the northward range shift.

**Figure 1:**
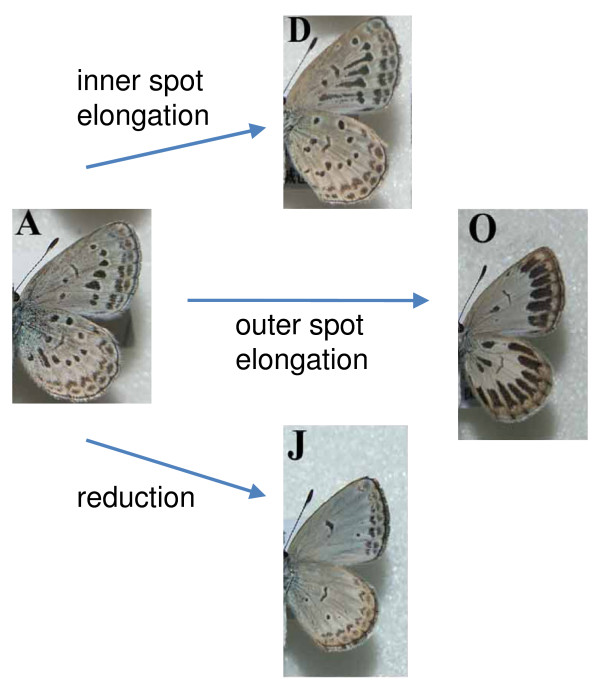
**Diverse wing spot pattern modifications found in colonizing populations of *Zizeeria maha *(modified from Otaki et al. 2010, Figure two)**.

Accompanying this colonisation, the pale grass blue has undergone an outbreak of wing pattern diversification. Three novel ventral wing patterns are recognizable with either inner spot elongation, outer spot elongation or reduction of both inner and outer spots (Figure 1). Novel phenotypes occurred at high frequencies in the new environments (10-15%), but were hardly ever seen across the main species range. These individuals showed no other obvious phenotypic changes, and the response was not similar to general stress responses seen in other butterflies [3,4]. A possible explanation of this diversity is the breakdown of developmental canalisation due to cooler winter temperatures in more northerly habitats. This is plausible as the pale grass blue butterfly typically does not experience temperatures less than 12°C across its largely tropical, southerly range. To investigate this, Otaki et al. [1] exposed pupae derived from the eggs of females caught in a southern population to cold-shock (6°C) and found this generated all three novel spot patterns. They also found a dose-response, with more novel forms developing after longer exposure to cold shock (10, 15 or 20 days).

Phenotypic plasticity has been proposed to be important in colonization and persistence in new environments [5-7]. Plasticity in a trait can reduce the difference between the mean phenotype of a recently-colonized population and the new environmental optimum, enabling the population to persist and so have sufficient time to adapt to the change in environment. Conversely, it could drive the mean phenotype away from the environmental optimum. In either case, plasticity increases the standing genetic load, by increasing phenotypic variance around the optimum. In the pale grass blue butterfly, the most obvious effect of colder temperatures in the northern environment is to vastly increase variation in wing spot patterns [1]. Whether there is a net benefit or loss in fitness associated with this is unclear. Similar responses to cold shock were seen with pupae derived from females caught in the northern range. However the eclosion rate was higher and the induction of wing pattern variation was less. This hints at phenotypic plasticity enabling individuals to gain a foothold outside the normal range, followed by local adaptation to more extreme temperatures and then the re-emergence of canalization to control variation in wing patterns.

Another feature of the northern population is that the novel wing spot patterns appear in summer butterflies. These could not have been exposed to lower temperatures as pupae, so temperature can't be the sole explanation. To investigate this further, Otaki et al. [1] undertook artificial selection for outer spot elongation under laboratory conditions. To simulate northern conditions, pupae were subjected to temperature shock during development. Otaki et al. observed a quick evolutionary response to selection, with over 80% of individuals developing modified wing patterns by the fifth generation. Elongated wing spots then started to appear without cold shock, with more than 40% of individuals showing novel wing spots without cold treatment by the tenth generation. This appears to be an example of "genetic assimilation" as proposed by C H Waddington in the 1940s, which proposes that exposure to a novel environment causes the expression of previously hidden genetic variation, which is then "assimilated" and expressed even in the absence of the novel environmental stimulus [8,9]. Such a process could underlie the appearance of butterflies with elongated spots in summer populations across the northern distribution of the pale grass blue which would not have been exposed to low temperatures.

For this explanation to make sense, selection needs to have favoured novel spot patterns in colonizing butterflies. Little is known about natural or sexual selection on ventral spot patterns in the pale grass blue, so this remains somewhat hypothetical. However, recent theoretical work has shown that sexual selection can enhance the effect of random genetic drift and act to diversify mate preferences and sexual colour patterns [10]. A change in mate preference could change the adaptive value of elongated spots from maladaptive in the main range, to adaptive in the new environment.

Another, perhaps more plausible, explanation is that selection favours traits in the novel environment that are genetically correlated to developmental control of wing patterning. It seems likely that there has been local adaptation to the novel colder environmental conditions in the colonizing population. Could such traits be genetically correlated to wing pattern traits? Unfortunately it is not clear from Otaki et al.'s study whether artificial selection on wing spots caused correlated genetic change in adaptation to cold shock, which would be indicative of an underlying genetic correlation. Pupal survival rate under temperature shock increased through time in the selection line, as predicted under this hypothesis. But there was no appropriate control. We need to know the degree of adaptation (i.e. pupal survival rate) of a population held under the same conditions of cold shock but without artificial selection on wing spots. Moreover, there was only a single replicate of artificial selection in Otaki et al.'s study; several would be needed to exclude random effects.

Certain types of phenotypic plasticity enable an organism to tolerate environmental variation without experiencing a major reduction in fitness in different environments. However environmental variation outside of the range usually experienced by an organism can generate novel phenotypic responses, which have not been previously shaped by selection [5], and may also reflect the expression of previously hidden genetic variation. Poleward range shifts provide an interesting natural system in which to study this phenomenon. Selection for canalization of developmental traits also makes these interesting candidates for responding in a novel manner to extreme environmental cues [8]. It would be interesting to see whether increased phenotypic variance is a common pattern in other species' range expansions, and whether there are underlying correlated genetic effects similar to those seen in wing patterning in the pale grass blue butterfly [1]. The unpredictability of the effects on fitness of such phenotypic change makes it hard to predict how such novel variability impacts on population persistence. Additional empirical examples, such as that presented by Otaki et al. [1], are necessary to explore the importance and generality of this interesting phenomenon, as is a more detailed understanding of the fundamental links between traits that lie within gene and developmental networks.

## Authors' contributions

JB, JRB and AP wrote the commentary. All authors read and approved the final manuscript.
